# 4-methylumbelliferone Prevents Liver Fibrosis by Affecting Hyaluronan Deposition, FSTL1 Expression and Cell Localization

**DOI:** 10.3390/ijms20246301

**Published:** 2019-12-13

**Authors:** Irina N. Andreichenko, Alexandra A. Tsitrina, Alexander V. Fokin, Adelya I. Gabdulkhakova, Dmitry I. Maltsev, Grigorii S. Perelman, Elena V. Bulgakova, Alexey M. Kulikov, Arsen S. Mikaelyan, Yuri V. Kotelevtsev

**Affiliations:** 1 Center for Neurobiology and Brain Restoration, Skolkovo Institute of Science and Technology, Skolkovo, 143025 Moscow, Russia; nikolavna.88@mail.ru (I.N.A.); Adelya.gabdulkhakova@skolkovotech.ru (A.I.G.); perelman.greg@gmail.com (G.S.P.); 2 Koltzov Institute of Developmental Biology of Russian Academy of Sciences, 26 Vavilov Street, 119334 Moscow, Russia; sashulka.s@gmail.com (A.A.T.); bifurc8@gmail.com (A.V.F.); mal-dima@yandex.ru (D.I.M.); elvbulg@gmail.com (E.V.B.); amkulikov@gmail.com (A.M.K.)

**Keywords:** liver fibrosis, 4-methylumbelliferone, hyaluronan, hyaluronan synthase, FSTL1

## Abstract

4-methylumbelliferone (4MU) is an inhibitor of hyaluronan deposition and an active substance of hymecromone, a choleretic and antispasmodic drug. 4MU reported to be anti-fibrotic in mouse models; however, precise mechanism of action still requires further investigation. Here we describe the cellular and molecular mechanisms of 4MU action on CCl_4_-induced liver fibrosis in mice using NGS transcriptome, Q-PCR and immunohistochemical analysis. Collagen and hyaluronan deposition were prevented by 4MU. The CCl_4_ stimulated expression of Col1a and αSMA were reduced, while the expression of the ECM catabolic gene Hyal1 was increased in the presence of 4MU. Bioinformatic analysis identified an activation of TGF-beta and Wnt/beta-catenin signaling pathways, and inhibition of the genes associated with lipid metabolism by CCL_4_ treatment, while 4MU restored key markers of these pathways to the control level. Immunohistochemical analysis reveals the suppression of hepatic stellate cells (HSCs) transdifferentiation to myofibroblasts by 4MU treatment. The drug affected the localization of HSCs and macrophages in the sites of fibrogenesis. CCl_4_ treatment induced the expression of FSTL1, which was downregulated by 4MU. Our results support the hypothesis that 4MU alleviates CCl_4_-induced liver fibrosis by reducing hyaluronan deposition and downregulating FSTL1 expression, accompanied by the suppression of HSC trans-differentiation and altered macrophage localization.

## 1. Introduction

Extracellular matrix (ECM) deposition is a natural reparative response to the liver injury caused by chemical toxins or viral infections. Fibrosis occurs as an excessive formation of ECM resulting in the subsequent growth of connective tissue in the affected areas. Collagen 1 and 3 are the major components of the fibrous formations in the liver. They are produced by myofibroblasts, which originate predominantly from the hepatic stellate cells (HSCs), pericyte like cells expressing desmin and glial fibrillary acidic protein (GFAP). In the quiescent state HSCs are uniformly spread throughout the liver parenchyma, providing storage for retinol. Upon activation by profibrotic cytokines, like PDGF and TGF-β peptides, HSCs undergo transdifferentiation to myofibroblasts characterized by the expression of alfa smooth muscle cell actin (αSMA) and vimentin. 

Activated HSCs lose retinol, migrate to the sites of injury, and deposit collagen fibers [1]. Resident macrophages (Kupffer cells) and blood-born, monocyte-derived macrophages play an important role in HSC activation and ECM turnover, providing both pro fibrotic and anti-fibrotic influence on different stages of fibrogenesis [2].

The initial stages of fibrosis caused by alcoholic and non-alcoholic fatty liver disease, hepatitis B and C, may be reversed after the elimination of pathogenic factors; however, if the injury persists, the liver fibrosis progresses through several stages to cirrhosis, characterized by impaired organ function, portal hypertension and malignant transformations, which require complex and expensive therapies [3].

At present there is no pharmacological treatment of liver fibrosis. Hence, the search for therapeutic targets and potential drugs is of great importance.

The naturally-occurring coumarin, 4-methylumbelliferone (4MU) is the active component of hymecromone, a widely used choleretic and antispasmodic drug. It is considered to be an inhibitor of hyaluronan deposition via its depletion of cellular UDP-GlucUA [4] and downregulation of HAS2 and HAS3 expression [5], however its direct action on the enzymatic activity of HAS was not demonstrated. 4MU was shown to significantly reduce the development of fibrosis in mice caused by a transplanted hepatocellular carcinoma cell line in the presence of thioacetamide. The action of 4MU was associated with the reduced deposition of hyaluronan (HA), the major glycosaminoglycan of ECM, in vivo. 4MU also prevented HSC activation in vitro [6]. However, the cellular mechanism of 4MU action on liver fibrosis in vivo has not been investigated.

Using immunohistochemical staining and confocal microscopy, we analyzed the mechanism of action of 4MU on cellular processes during CCl_4_-induced liver fibrosis in mice. Herein we confirm that 4MU suppresses HA deposition, as well as alleviating fibrous formation and collagen deposition. Most interestingly, 4MU downregulates expression of follistatin-like protein 1 (FSTL1), an important cytokine of the TGF-beta signaling pathway, induced by the organ injury. 

We also have demonstrated reduced numbers and altered localization patterns of activated HSCs along the damaged regions of the liver lobes. This was associated with the altered localization and increased catabolic activity of macrophages.

4MU not only prevented fibrosis when administered together with CCl_4_, but also accelerated the process of regeneration after this intoxication with CCl_4_ was canceled.

Our findings suggest that hymecromone can be re-purposed as an anti-fibrotic drug in the early stages of liver disease. It also provides the starting point for the development of new, small molecules affecting hyaluronan synthesis and FSTL1 signaling, which may have broader applications.

## 2. Results

### 2.1. 4MU Treatment Reduces Fibrosis and Prevents Hyaluronan Deposition in CCl_4_-Treated Mice

**Figure 1:**
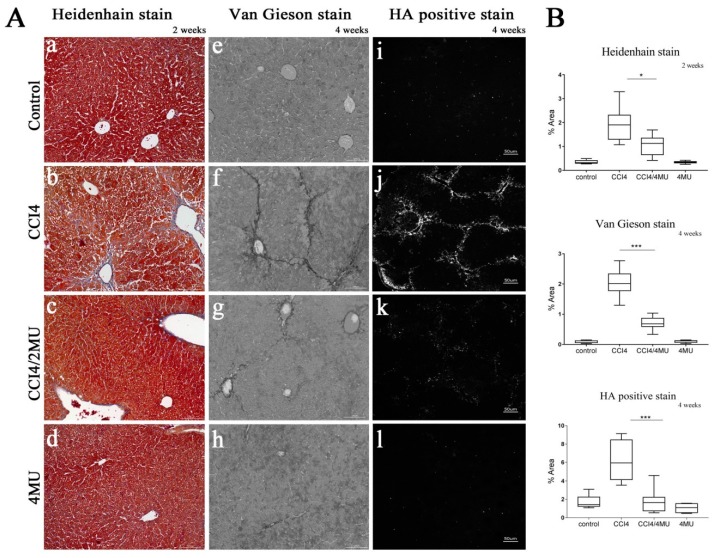
Effect of 4-methylumbelliferone (4MU) on collagen and hyaluronan deposition and fibrotic scar formation. (**A**) Heidenhain and Van Gieson stains for collagen and hyaluronan binding protein staining for hyaluronan deposition after two weeks and four weeks of CCl_4_ treatment. Scale bar 100 um (**a**–**h**), scale bar 50 um (**i**–**l**). (**B**) Morphometric quantification of collagen and hyaluronan deposition * *p* <0.05; *** *p* < 0.001. Data presented as mean ± standard deviation (SD).

Repetitive treatment with CCl_4_ resulted in liver fibrosis characterized by pericentral and periportal fibrous depositions bridging portal veins. The patterns of fibrous deposition were similar in Van Gieson and Heidenhain staining. Observed changes correspond to F2 stage fibrosis according to the Metavir fibrosis scoring system (Figure 1A(b,f)). Daily 4MU per os (by mouth) treatment of mice subjected to CCl_4_ inhalation reduced the area on the liver sections stained for connective tissue by 50% at the two weeks point (Figure 1B) and by 70% at the later four weeks point (Figure 1A(c,g),B). Mice well tolerated the 4MU given by gavage. There was no difference in behavior, and they gained weight similar to untreated Balb/c controls.

4MU ameliorated fibrosis as well, if given two weeks after the beginning of CCl_4_ inhalation, and this effect persisted either CCl_4_ treatment was continued or stopped for the last two weeks (Figure S2).

Treatment with 4MU dramatically reduced the deposition of hyaluronan. We observed intense hyaluronan staining, associated with the areas of fibrous formation in CCl_4_-treated animals (Figure 1A(j)), which was completely abolished at the four weeks point in 4MU-treated animals (Figure 1A(k)).

### 2.2. 4MU Prevents Transdifferentiation of HSCs to Myofibroblasts and Alters Localization Pattern of Myofibroblasts and Macrophages during Fibrosis Induced by CCl4 Injury

**Figure 2:**
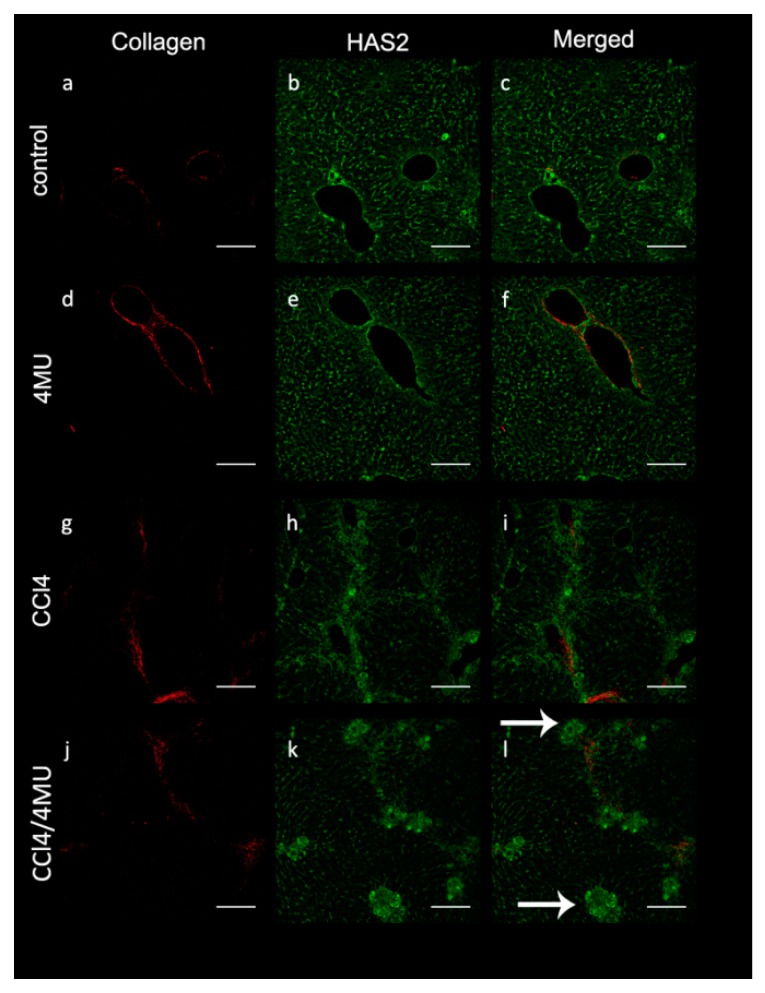
HAS2-positive cells accumulation around collagen fibers in CCL_4_-exposed animals and aggregate in clamps in the CCL_4_/4MU-treated group (see arrows). The second harmonic generation method was used for collagen detection. (a,d,g,j). HAS2 was detected immunohistochemically (b,e,h,k). Merged images (c,f,i,l), scale bar 100 um. Arrows indicates clamps of HAS2-positive cells.

**Figure 3:**
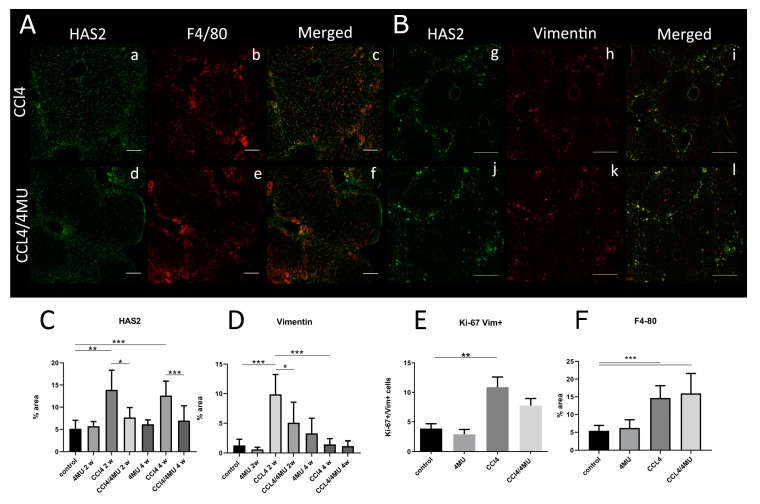
4MU reduces HAS2 expression by macrophages and activated hepatic stellate cells (HSCs) in CCl_4_-treated animals at two weeks. (**A**) Expression of HAS2 (a,d) on macrophages (F4/80; b,e), scale bar: 100 um. (**B**) Expression of HAS2 (g,f) by activated HSCs (Vimentin; h,k), scale bar: 100 um. Arrows indicate double-positive cells. Morphometric evaluation of HAS2 (**C**), Vimentin (**D**) at two and four week time points. Evaluation of Ki-67/Vim-positive cells (**E**) and F4/80 positively stained area and (**F**) at the two week time point. *** *p* < 0.001, ** *p* < 0.01, *p < 0.05.

Reduced collagen deposition in CCL_4_/4MU-treated animals was paralleled by changes of immunohistochemical staining with anti-HAS2 antibodies (Figure 2). In CCl_4_-treated animals HAS2+ cells were aligned with the septa (acinus zone 1) and formed continuous stretches. However, these stretches were disrupted CCL_4_/4MU-treated animals with HAS2-positive cells gathered in clamps at the edges of the septae (Figure 2, see arrows). Exposure to CCl_4_ increases the area occupied by HAS2-positive cells, which also bear markers of both activated HSC (vimentin) and macrophages (F4/80) (Figure 3A,B). 

Morphometric analysis revealed that CCl_4_ exposure significantly upregulates HAS2 expression at 2 and 4 weeks (Figure 3C). 4MU treatment attenuated this effect at both time points.

Vimentin, the marker of activated HSCs, was nearly absent in the healthy controls and 4MU-treated groups (data not shown). CCL_4_ exposure for two weeks markedly increased vimentin staining. At four weeks of CCL_4_ treatment, we observed a reduction of the vimentin-stained area to the control level. This data indicates the maximal activation of HSCs at two weeks and possible deactivation or senescence at four weeks. 4MU treatment significantly decreased the area, occupied by the Vim+-activated HSC (Figure 3B,D) at two weeks. We also measure the proliferation of HSCs (Figure S3) using ki-67 staining. We did not observe any significant decrease in HSCs proliferation at the two week time point (Figure 3E).

F4/80-positive cells spread uniformly in liver parenchyma in control and 4MU-treated animals (data not shown). After CCL_4_ exposure, we observed the accumulation of macrophages in fibrous septae areas, surrounded by cells migrating from liver parenchyma (Figure 3A).

In CCL_4_/4MU-treated animals, the F4/80-positive area was unchanged compared to our CCl_4_-treated group. (Figure 3A,E), but the localization pattern was changed with F4/80-positive cells lined predominantly in the septae area. 

For detail characterization of 4MU effect on fibrogenesis, we evaluated the Tresholded Manders’ coefficients for the colocalization of HAS2 on macrophage (F4/80) on HSCs (GFAP). This approach allowed us to determine relative changes in HAS2 expression on macrophages and HSCs (Table 1).

4MU alone significantly increases the proportion of F4/80 which colocalized with HAS2 (*p* < 0.001) comparing with control. CCL_4_ treatment caused a significant increase fraction of HAS2 (*p* < 0.05), expressed on macrophages, compared to the control group. CCL_4_/4MU treatment reduced the fraction of HAS2 to the control level (*p* < 0.01).

4MU reduces the fraction of HAS2 presented on HCSs (*p* < 0.001). CCL_4_ induces the same effect (*p* < 0.01). CCL_4_/4MU slightly increases the association between GFAP and HAS2, but HAS2 fraction did not change significantly.

In the CCL_4_/4MU group, macrophages demonstrated the decrease of HAS2 expression in comparison with the CCL_4_ group. In the same group, HSCs presented the same level of HAS2 expression as in the CCl_4_-treated group. Quantification of the F4/80-stained area (Figure 3F) did not show any difference between these CCl_4_- and CCL_4_/4MU-treated groups, thus the number of macrophages was unchanged by 4MU treatment. This data indicates the reduction of HAS2 expression by 4MU specifically in macrophages and not in HSCs. 

Thus, in fibrosis settings, 4MU treatment (CCl_4_/4MU group) causes a dramatic reduction of HAS2 expression on macrophages. HSCs did not change their HAS2 expression, but demonstrated a reduction of cell number in our CCL_4_/4MU-treated group compared with CCl_4_ treatment only.

### 2.3. Hepatic Transcriptome Analysis of 4MU Antifibrotic Activity

Expression profiling was performed to identify potential pathways and key regulatory genes affected by 4MU in the CCl_4_ model of fibrosis.

**Figure 4:**
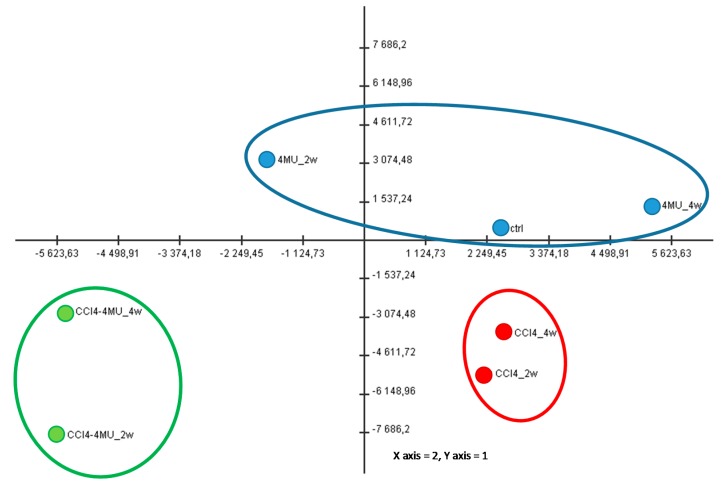
Of PC1 and PC2 scores for experimental groups. Principal components analysis (PCA) was obtained by values of 2847 differentially-expressed genes selected with one-way Analysis of Variance (ANOVA). The first two components cover two thirds of the variability of expression of all analyzed genes. X–axis–PC1, Y–axis– PC2.

Hepatic levels of RNA expression were measured by high throughput sequencing in normal and CCl_4_ fibrotic mice treated and not treated with 4MU at two and four week time points. Transcriptome data analysis revealed 2847 differentially expressed genes in seven experimental groups depicted in Figure 4. The principal component analysis showed that 72% of gene expression variability was covered by the first two components (data not shown). Distribution of sample groups in the space of these two components revealed three big experimental cohorts. 4MU treatment alone did not show any significant differences in gene expression compared to control, and these three experimental groups fall into the first cohort. The CCL_4_-treated group was separated from the control and CCL_4_/4MU-treated samples, and formed the second cohort. CCL_4_/4MU-treated samples were significantly different from the control and CCL_4_-treated group, and were assembled into the third cohort (Figure 4).

**Figure 5:**
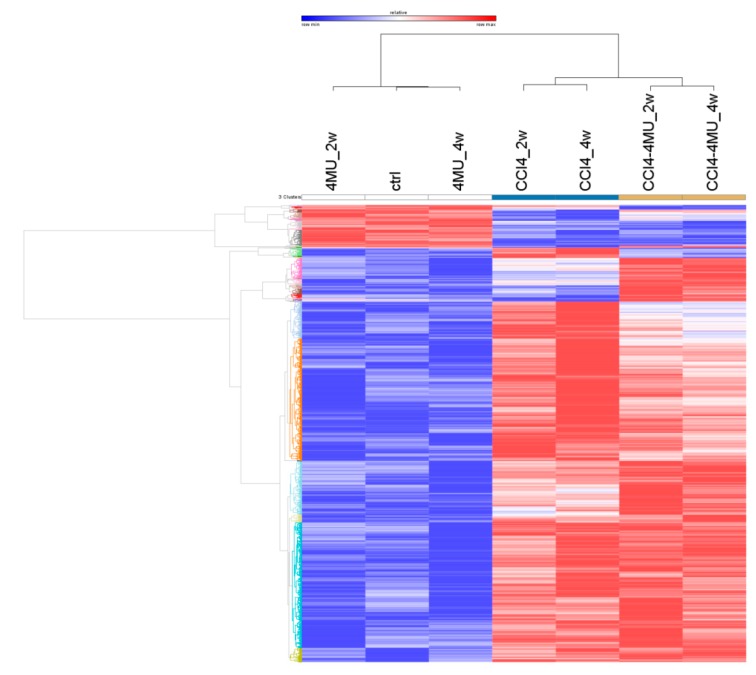
Heat map of differential expression of 2847 genes in seven experimental groups.

Cluster analysis of differentially-expressed genes revealed two big clusters according to the fold change difference of gene expression across the whole experiment (Figure 5). The first cluster contains genes, which abundantly expressed in the first cohort, and were downregulated in the second and third cohorts. Genes, the expression of which was upregulated in the second and third cohort compared with first one, fall into the second cluster. Both clusters contain three smaller ones that described the different effects of CCl_4_ and CCl_4_/4MU action onto gene expression.

Individual genes expression data is represented in Supplementary RNA NGS data file. In CCl_4_-treated mice with histologically documented fibrosis, collagens genes Col1a1, Col1a2 were drastically upregulated and suppressed by 4MU treatment at two and four weeks, without being affected by 4MU treatment alone. 

Analysis of the CHEA 2016 database revealed overlap with genes regulated by RXR, PPARa and LXR in all experimental samples, being downregulated by CCl_4_ and up regulated in CCL_4_/4MU treatment to control level.

It is of interest that we also have observed a dramatic upregulation of cytochrome Cyp3a11, the main target of PXR (Pregnane X receptor) [7]. Cyp3a11 was expressed at a very high level in the control, suppressed two fold by CCl_4_ treatment and upregulated 3–6-fold by CCL_4_/4MU. This puts PXR as a potential target for 4MU. Indeed, agonists of PXR equol, genistein and daidzein have striking structural similarity with 4MU [7].

In CCl_4_-treated samples we observed significant upregulation in genes involved in cell cycle and cell division, ECM-receptor interaction and focal adhesion signaling pathways. We found significant overlap between (myo)-fibroblasts, Kupffer cells, hepatic stellate cells and the macrophages specific gene expression signature and CCL_4_-upregulated genes.

CCL_4_ exposure downregulates expression of the genes involved in lipid metabolism in the liver. We observed significant enrichment of genes involved in Cholesterol and Fatty acids metabolism, including betta-oxidation of fatty acids. 

Amino acids metabolism, retinol metabolism and coagulation cascade genes were also affected. Hepatocytes were the primary cell types associated with such changes.

Cotreatment of 4MU and CCL_4_ significantly upregulates genes involved in cholesterol and retinol metabolism, amino acid metabolism, CoA-biosynthesis and peroxisomal oxidation. The main affected cell type was hepatocytes. Downregulated genes were involved in Focal adhesion, Insulin Signaling, IL-5 signaling and Notch signaling pathways. The main affected cell type was fibroblasts.

### 2.4. 4MU Reduces the Expression of Key pro Fibrotic Genes and Increases the Expression of Hyaluronidases

**Figure 6:**
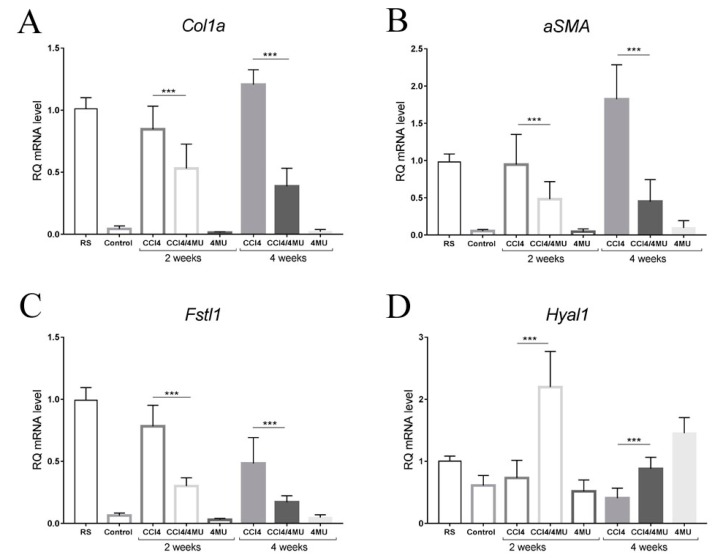
Expression of key profibrotic genes (Col1A and aSMA), Fstl-1 and Hyal1 measured by Q-PCR. (**A**) 4MU downregulated Col1a expression on 30%/70% on 2/4 week time points correspondently. (**B**) Stimulated expression of *aSMA* was downregulated up to 70% by 4MU treatment at two weeks and four weeks. (**C**) Treatment by 4MU caused a significant decrease in the *FSTL1* mRNA level in both experimental time points. (**D**) *Hyal1* mRNA levels were upregulated in CCI_4_/4MU, compared to the CCI_4_-treated group in both time points. Data present as mean ± SD *** *p* < 0.001. RS–reference sample.

*Col1a* m RNA expression measured by Q-PCR was significantly upregulated in mice treated with CCl_4_ for two and four weeks than in control mice. 4MU downregulated Col1a expression on 30% /70% on 2/4 week time points correspondently (Figure 6A). The marker of myofibroblasts *aSMA* was virtually absent in healthy liver and was maximally stimulated by CCl_4_ injury at the four weeks point. Stimulated expression of *aSMA* was downregulated up to 70% by 4MU treatment at two weeks and four weeks (Figure 6B). 

According to our transcriptome data, the *FSTL1* gene was identified as one of the most upregulated genes in the CCl_4_-treated group. Its expression was at a very low level in healthy mice and was increased dramatically under CCl_4_ exposure, reaching its maximum at two weeks. At the four weeks point, the FSTL1 mRNA level was less than at two weeks, but still higher than in control. Treatment by 4MU caused a significant decrease in the *FSTL1* mRNA level in both experimental time points (Figure 6C).

Interestingly, among all investigated genes only *Hyal1* mRNA levels were upregulated in CCI_4_/4MU, compared to the CCI_4_-treated group in both time points (Figure 6D).

**Figure 7:**
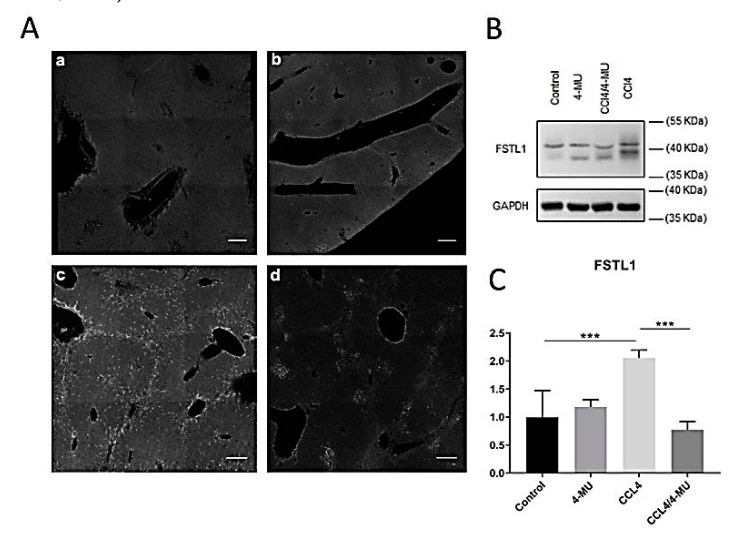
Evaluation of 4MU effects on Fstl-1 protein level during CCl4 induced fibrosis. (**A**) Immunohistochemical characterization of Fstl-1 in two weeks liver samples: a—control group, b— 4MU-treated group, c—CCl_4_-treated group, d—CCL_4_/4MU treated group, scale bar: 100 um. (**B**) Western-blot analysis for FSTL-1 at a two-week time point. (**C**) Quantification Fstl-1 immunopositive bands, normalized on the glyceraldehyde 3-phosphate dehydrogenase (GAPDH) level. Data represented as mean ± SD *** *p* < 0.001.

Immunostaining and Western blot analysis confirmed the inhibiting effect of 4MU on FSTL-1 expression during the progression of fibrosis (Figure 7B,C). Immunohistochemical staining demonstrated specific patterns of Fstl-1 expression. In CCl_4_-treated groups (Figure 7A(c)) Fstl-1 repeated the pattern of Coll1, HAS2, Vimentin F4/80, and stretches along the fibrous septae. 

FSTL-1-positive cells aggregated in clamps in CCL_4_/4MU-treated mice. In untreated mice and in healthy mice treated with 4MU, the Fstl-1 signal was relatively low and uniformly spread in tissue parenchyma (Figure 7A(a,b)).

## 3. Discussion

In this study we describe the potential therapeutic effect of 4MU on the progression of CCl_4_-treated liver fibrosis in mice associated with the inhibition of HA deposition and identify possible pathways that may be involved in its therapeutic action. On the cellular level 4MU affects the activation and localization of HSCs and macrophages. 4MU polarizes macrophages towards the regenerative phenotype, reducing HAS2 expression and enhancing hyaluronidase expression. We also identify FSTL1 protein, an important cytokine of the TGF-beta pathway as a possible regulator of these processes.

4MU does not protect hepatocytes from cytotoxic damage. ALT/AST levels were elevated at both time points (data in Figure S1) in CCl_4_-treated animals, and were not significantly different in CCL_4_/4MU-treated mice. Hence, the anti-fibrotic effect of 4MU cannot be explained by possible anti-oxidant protective effect of 4MU on hepatocytes [8].

4MU rather influences ECM metabolism and fibrous septa formation through its effect on macrophages and HSCs. Importantly, it reduces the expression of the Col1a major form of collagens contributing to ECM remodeling in liver fibrosis. 

The Balb/c strain was chosen because it develops more significant liver fibrosis caused by CCl_4_ than the C57BL/6 strain due to the prevalence of the Th2-type response [9]. The CCl_4_ inhalation model which we used in this study provided highly reproducible peri-central fibrosis with centro-central septa and porto-portal septa formation, and the region first affected by inhalation route of CCl_4_ Stronger fibrous septae was developed across acinus zone 1 between portal triads with weaker stretches extended to the central vein. This type of fibrous deposition was described earlier in methodological studies comparing different protocols of CCl_4_ application [10]. Our protocol of inhalation was moderate, and did not result in cirrhosis and cyst formation at two and four weeks points, as described in the CCl_4_ injection model [11]. The area affected by fibrosis can be quantified with equal accuracy using Mallory, Van Gieson or Heidenhain staining [12]. 

4MU is known to reduce HA deposition presumably through depletion of intracellular UDP–glycoside stocks [5]. In our studies HA was virtually undetectable in the healthy liver by confocal microscopy using fluorescently labeled-HABP. After CCl_4_ treatment, abundant HA depositions appear across the areas stained for fibrous formation by histological techniques. 4MU reduced the deposition of HA almost to the negligible background level presented by the healthy liver. Previous studies revealed the role of HA in several types of fibrosis reviewed in [13] and in provisional matrix formation [14]. Increased plasma HA levels are a well-known biomarker of liver fibrosis in chronic hepatitis C [15]. Recently it was shown that a low dose (20 mg/kg BW) of 4MU alleviated bleomycin-induced pulmonary hypertension hyaluronan deposition, but not lung fibrosis in mice, although the mechanism of its action was not investigated [16]. We consider that a dramatic reduction of HA levels by 4MU is essential for the amelioration of fibrosis. 

The first observation of the anti-fibrotic action of 4MU in the liver was published by the Guillermo Mazzolini group in 2012 [17]. The authors established an orthotopic model of HCC (transplanted Hepa129 cell line) in mice with advanced fibrosis induced by thioacetamide (TAA). 4MU applied at 20 mg/kg IP was investigated for its anti-tumor properties, and also unexpectedly diminished fibrosis and prevented cyst formation in TAA-treated mice. The drug also caused apoptosis of activated HSCs in vitro. Later the same group has shown that oral administration 4MU (400 mg/kg daily) inhibited tumor growth, decreased IL-6 production in Kupffer cells, and reduced cell migration and angiogenesis in the HCC+TAA model [6]. While this manuscript was in preparation, a paper was published showing an amelioration of CCl_4_-induced liver fibrosis in rats by umbelliferone (UMB), a close analog of 4MU, at three different per os doses (25, 50, 100 mg/kg). The effect of 4MU was associated with the upregulation of PPRα, attenuation of oxidative stress, inflammation and TGFβ1/Smad3 signaling [18]. Reported pharmacological activities of UMB are nociceptive, anti-inflammatory and anti-hyperglycemic [19]. Unfortunately, the effect of UMB on hyaluronan synthesis and deposition was not studied.

Immunohistochemical staining with markers of quiescent and activated HSC (Vimentin) and macrophages (F4/80) revealed the effect of 4MU on the activation and migration of HSC and macrophages.

Interestingly, the analysis of Manders coefficient for paired markers indicated that CCl_4_ treatment induces HAS2 expression on macrophages which is reduced by 4MU treatment, with the number of cells remaining unaffected. At the same time, the main effect of 4MU on the reduction of HAS2 expression contributed by HSCs was through the reduction of the cell numbers, rather than through reduced expression of the enzyme by the individual cells.

Analysis of the CHEA 2016 database revealed overlap with genes regulated by RXR, PPARa and LXR in all experimental samples, being downregulated by CCl4 and upregulated with 4MU treatment to the control level. These nuclear steroid receptor-type transcription factors act as homo- and heterodimers, and it is worthy to suggest that 4MU acts as a ligand of one of those. We also have found several pathways affected by 4MU, which downregulates different stages of mitosis, including synthetic phase elongation and initiation factors), spindle formation, histone dissociation and transcription. These findings concord with the mild cytostatic action of 4MU which we and others observe in cell culture experiments [20].

Here we demonstrate for the first time that the expression signatures, associated with genes, involved in lipid metabolism, are affected by 4MU after CCl_4_ treatment. This might be a potential route for regulation of HAS activity, as it depends on its lipid microenvironment [21]. Unfortunately we have not detected the expression of HAS enzymes, which is not surprising, given their very low level of expression based on our Q-PCR data. An alternative explanation of the reduced expression of the genes involved in lipid metabolism might be milder liver injury and reduced hepatic stellate cell activation.

FSTL1, widely expressed secreted glycoprotein, homologous to follistatin and SPARC (known as TSC-36) was cloned from a mouse osteoblastic cell line as a TGF-beta inducible gene [22]. Recently FSTL1 attracted attention as a possible modulator of pathological processes and as a potential drug target, reviewed in [23]. 

According to our transcriptome data, FSTL1 was identified as one of the most upregulated genes in the CCl_4_ model. We concentrated specifically on the dramatic upregulation of FSTL1 by CCl_44_ treatment and its suppression by 4MU. In this study, we report the first time that small molecule, 4MU, which downregulates mRNA level and protein expression of FSTL1 in the settings of family (also experimental liver fibrosis conditional knockout of FSTL1 in fibroblasts demonstrated its essential role in cardiac fibroblast activation during cardiac muscle repair after ischemic injury) [24]. FSTL1 is essential for keratinocyte migration during wound healing [25].

Several papers validated FSTL1 as a promising drug target for the treatment of fibrosis. Targeting FSTL1 with a specific antibody in vivo ameliorated bleomycin-induced lung fibrosis in mice, similar to FSTL1 genetic insufficiency in transgenic mice heterozygous for the FSTL1 knockout allele [26]. Further studies on this model revealed that FSTL1 affects lung fibroblast differentiation, proliferation, migration and invasion through p38 and JNK signaling [27].

FSTL1 came out of the comparative screen of the genes activated in renal and liver fibrosis. Knockdown of FSTL1 in vivo using specific siRNA-reduced *Col1a1* mRNA expression, collagen deposition and macrophage accumulation, but surprisingly increased myofibroblast density in the tissue. Interestingly, knockdown of HAS2 mRNA had a very similar effect if compared to the one of FSTL1 KD [28].

Studies in primary cultures of HSCs isolated from mouse liver place FSTL1 downstream TGFb1 activation and upstream regulator of αSMA and Col1a1 production. The knockdown of FSTL1 with siRNA in HSCs stimulated with TGFb1 reduced αSMA and Col1a1 expression almost to the background levels. TGF-b1-induced phosphorylation of Smad3 also was reduced [29].

Elevated levels of FSTL1 were found in plasma from patients with silicosis and in lungs of mice with silica-induced fibrosis. In mice Fstl1 promoted fibrosis via activation of proinflammatory NOD-like receptor family in macrophages. Haploinsufficiency of *Fstl1* as well as a neutralizing antibody to FSTL1 protected from silica-induced lung injury in mice in vivo [30].

A recent extensive study demonstrated that conditional knockout of has2 HSCs reduced liver fibrosis in mice [31]. It also shows that HAS2 was upregulated by transforming growth factor-β through Wilms tumor 1 to promote transdifferentiation of HSCs towards myofibroblasts. RNA-Seq analysis was performed on the fibrotic livers of HSC-specific HAS2, CD44 and TLR4 conditional knockout mice. This paper proposed activation of profibrotic processes by low molecular weight hyaluronan produced by HAS2 localized on HSCs and particularly activated HSCs via CD44, Toll-like receptor 4 (TLR4), and Notch1. It was shown that 4MU had no further antifibrotic effect over HAS2 or notch1 deficiency. Our paper complements this data with RNA-seq analysis of 4MU-treated mice with CCl_4_-induced fibrosis. Downregulation of FSTL1 that we demonstrate here is correlated with possible downregulation of Notch1, demonstrated by its knockout in the proteomic screening [32]. We also identify nuclear RXR, PPARa, LXR and PXR receptors as possible targets for 4MU. It was not clear from this study whether inhibition of HA synthesis by 4MU affects these pathways. Our data support the action of 4MU through Wnt/beta-catenin, and do not corroborate the published data on Notch1 involvement. 

## 4. Materials and Methods

### 4.1. Animal Model

All regulated animal procedures were conducted following the Russian Academy of Science Guidelines for Animal Experimentation and are approved by the Institute of Developmental Biology RAS Ethics committee. Balb/c 8-week old female mice were maintained in the animal facility of the Institute of Developmental Biology RAS according to GLP requirements at room temperature, controlled light/dark cycle and relative humidity 55%–65%. All groups were provided with free access to the standard diet and water. Animal experiments were carried out following the recommendations of the IDB ethics committee. Mice acclimatization to the conditions lasted 2–3 days before the experiments start.

Liver damage was induced by exposing mice to CCl_4_ vapors in a closed 5 L glass chamber. For that, 80 uL of pure CCl_4_ was evaporated from a filter paper for 15 min at room temperature. This inhalation procedure was repeated two times a week for two or four weeks.

4MU was purchased from Sigma-Aldrich (Cat # M1381, St. Louis, MO, USA). 4MU dose selection was based on a recently published pharmacokinetic study [33]. 4MU was prepared by mixing with 0.5% methylcellulose which was given per os by gavage at a concentration of 600 mg/kg daily. Control mice were gavaged in the same way with 0.5% methylcellulose slurry. The administration of 4MU was started two days before the first inhalation and lasted for two or four weeks. The typical experiment consisted of six groups of animals (*n* = 8), untreated control; healthy 4-treated MUCCl_4_-treated and CCL_4_/4MU-treated. At the end of two and four weeks of treatment, animals were terminally anesthetized with 5% isoflurane and killed by fast exsanguination followed by cervical dislocation. Liver samples were collected for RT-PCR analysis, histological examination and Western blot. Separate groups of animals were used for immunohistochemical examinations and confocal microscopy.

### 4.2. Blood Serum Collection and ALT/AST Measurement

Whole blood was sampled from the left ventricle after culling the animal. Blood samples were left for coagulation for 1 h on ice. Serum was purified by centrifugation for 40 min at 3000 rpm and frozen at −20 °C for storage. The ALT/AST level was measured by the commercial biomedical analytical laboratory ChanceBio, Moscow.

### 4.3. Histology Examination

Freshly isolated liver samples were cut onto pieces 3 × 3 mm and fixed in 10% buffered formalin for 24 h. After extensive wash in diH_2_O, samples were dehydrated in isopropanol solutions with rising concentration from 70% to 100%, followed by two immersions in xylene and then being embedded in Histomix (Biovitrum, Russia) at 56 °C. Embedded tissue samples were sectioned by microtome at 5 µm slices and mounted onto SuperFrost glass slides. For Mallory, Van Gieson and Heidenhain staining, we used commercially-available kits (Biovitrum, Russia).

### 4.4. Immunohistochemical Analysis

For the confocal microscopy study we used separate groups of mice (*n* = 4–8). Mice were terminally anesthetized by 5% isoflurane inhalation and perfused through their left ventricle with PBS followed by 10% buffered formalin (Biovitrum, Russia) for 20 min. Liver lobes were cut off and additionally fixed in 10% buffered formalin for 2 hours at RT. After samples were washed by PBS and cut for 50 um slices by vibratome sectioning.

Vibratome sections were permeabilized in PBS-0.2% Triton X-100 for 1 h, followed by incubation with primary antibody for twodays at +4 °C. After several washes with PBS-0.2% Triton X-100, sections were incubated with appropriated secondary antibody for one night. Washed sections were mounted in TDE-mounting media and analyzed by laser scanning confocal microscopy. Images were taken by a Carl Zeiss LSM800 confocal microscope (Carl Zeiss, Germany) equipped with 25× oil immersion objective (LCI “Plan-Neofluar” 25×/0.8 Imm Corr Ph2 M27, Carl Zeiss), 488 nm, 561 nm and 640 nm wavelength diode-lasers were used for fluorochrome excitation. Mosaic frame Z-stack, 4 × 4 observation field in size, were scanned on each sample in multitrack mode with 2 um optical section step. 

Following primary and secondary antibodies were used: FSTL-1 (BAF1738, R&D System, 1:100); HAS2 (MBS9207356, MyBioSource, 1:300, San Diego, CA, USA); CD44 (LS-C150276-100, Life-Span Bioscience, 1:300, Seattle, WA, USA); GFAP (ab50738, Abcam, 1:100); Vimentin (PA1-16759, Invitrogen, 1:300, Carlsbad, CA, USA); Ki-67 (ab15580, Abcam, 1: 300, Cambridge, United Kingdom); F4/80 (ab186073, Abcam, 1:100).

For hyaluronan detection we used biotinylated hyaluronan binding protein: HABP (385911-50UG, Millipore, 1:100). Briefly, paraformaldehyde-fixed paraffin-embedded sections were dewaxed and rehydrated in xylene–alcohol and rinsed in diH2O and PBS. For negative control, bovine testis hyaluronidase treatment was used (100 ug/ml, 1h, 37 °C). Avidin-biotin blocking system (SP-2001, Vector laboratories, Burlingame, CA, USA) was used for blocking unspecific binding according to manufacturer’s recommendation. HABP (385911-50UG, Millipore, Burlington, MA, USA) was applied in PBS-0.2%Triton X-100 for one night, RT. After intensive wash with PBS, HABP signal was detected by Streptevidin-Cy3 (S6402-1ML, Sigma-Aldrich Co, St Louis, MO, USA), 1h, RT. Samples were mounted in Prolong Gold antifade mounting media (P36930, Thermo Fisher, Waltham, MA, USA).

Secondary Ab: Donkey anti-rabbit Alexa Fluor 488 (A-21206, Invitrogen, 1:1000); Donkey anti-Goat Alexa Fluor^®^ 555 (A-21432, Invitrogen, 1: 1000); Goat anti-Chicken IgY Alexa Fluor^®^ 546 (A-11040, Invitrogen, 1:1000); Donkey anti-Sheep Alexa Fluor^®^ 568 (A-21099, Invitrogen, 1: 1000); Alexa Fluor 647 Tyramide SuperBoost kit Streptavidin (B40936, Invitrogen).

For collagen detection we used the published method of second harmonic generation by collagen fiber [34]. Images were taken by Leica SP5 confocal microscope (Leica, Germany), equipped with HCX PL APO CS 20.0 × 0.70 IMM UV objective lens and Ti-Sappfire Mai-Tai multiphoton laser.

### 4.5. Colocalization Analysis

For estimation of colocalization between HAS2-F4/80 for macrophages and HAS2 and GFAP for HSCs we choose Manders’ Colocalization coefficient (MCC). For pared markers R and G, two different values of MCC derived, *M*_1_, the fraction of *R* in compartments containing *G* and *M*_2_, the fraction of *G* in compartments containing *R. M_1_* and M_2_ were calculated as:

$$M_{1}=\frac{\sum_{i}R_{i\;coloc}}{\sum_{i}R_{i}}$$

$$\;M_{2}=\frac{\sum_{i}G_{i\;coloc}}{\sum_{i}G_{i}}$$

The analysis was done using colocalization module of Imaris 7.4.2 software (Bitplane, Switzerland).

### 4.6. Library Preparation and Sequencing

PolyA mRNA was collected from pooled samples of experimental and control groups described above (*n* = 6 to 10 mice in group) with NEBNext Poly(A) mRNA Magnetic Isolation Module (New England BioLabs, Ipswich, MA, USA) and Illumina cDNA libraries were constructed using NEBNext Ultra II Directional RNA Library Prep Kit for Illumina (New England BioLabs) following the manufacturer’s protocol. cDNA libraries were sequenced using the NextSeq500 (Illumina, San Diego, CA USA) instrument. 33–41 million raw reads were obtained for each sample with a 75 bp read length.

### 4.7. Transcriptome Data Analysis

Mice transcriptomes in fastq file format were aligned in TopHat/TopHat2 software (available online: https://ccb.jhu.edu/software/tophat/index.shtml, accessed on 12 December 2019). The CLC Genomics Workbench 7.0.3 (CLC Bio, Denmark) was used for read trimming with the following parameters: “quality scores–0.05; trim ambiguous nucleotides–2; remove 5′-terminal nucleotides–1; remove 3′-terminal nucleotides–1; and discard reads below a length of 25”. The 93% of bases had scores as the Q30 for single reads. The high-quality reads were mapped on the Ensembl Mus musculus genome (Mus_musculus.GRCm38.94) as a reference using the same program. The mapping parameters included unique mapping only, insertion cost – 3, deletion cost – 3, mismatch cost – 2, similarity fraction – 0.94. For each gene, total gene reads (TGR) was determined as the sum of all the reads mapped on this gene.

Then gene expression was calculated for each group and differential expression of each pairwise contrast was identified using DeSeq (available online: https://bioconductor.org/packages/release/bioc/html/DESeq.html, accessed on 12 December 2019) and DeSeq2 (available online: https://bioconductor.org/packages/release/bioc/html/DESeq2.html, accessed on 12 December 2019) software. Differences were considered significant at values of *p* < 0.05. ANOVA and PCA were performed with MeV [35], taking into account the separation of all experimental samples into three groups Then we used permutation-validated principal component analysis (PCA), to determine which combinations of correlated expression-altering genes best differentiate the experimental groups. The Heat Map and the Venn diagram were generated with GENE-E [36].

GO Molecular functions, GO Biological processes KEGG, KEGG Pathways, WikiPathways and CHEA 2016 databases were used to annotate differences in molecular functions, biological processes and signaling pathways between experimental groups with Enrichr (http://amp.pharm.mssm.edu/Enrichr), Webgestalt (available online: http://webgestalt.org, accessed on 12 December 2019) and Pathway Studio (available online: http://elsevierscience.ru/products/pathway-studio, accessed on 12 December 2019) instruments.

Specificity to liver tissue was confirmed in ARCHS4 Tissues (Massive Mining of Publicly Available RNA-seq Data from Human and Mouse) atlas.

### 4.8. Q-PCR Analysis

Total RNA from fresh tissue samples was isolated by TRI reagent (Sigma-Aldrich, St. Louis, MO, USA) according to the manufacturer’s protocol. The concentration and quality of total RNA was determined by using the NanoDrop 2000 spectrophotometer (Thermo Fisher Scientific, Inc.) and electrophoresis. To eliminate genomic DNA contamination from RNA samples DNA-free™ DNase Treatment and Removal Reagents (Ambion) were used. 2 ug total RNA was used for cDNA synthesis by Mint-2 (Evrogen, Moscow, Russia) cDNA synthesis kit and random primers according to the manufacturer’s protocol. Evaluation of mRNA Expression Levels of target genes was measured by using qPCRmix-HS HighROX SYBR (Evrogen, Moscow, Russia) and Applied Biosystems StepONE Plus Real-Time PCR System (Thermo Fisher Scientific, Inc.). All data were presented as mean ± SD of three independent technical replicates and normalized to the level of glyceraldehyde 3-phosphate dehydrogenase (GAPDH) mRNA. As a reference sample, for the normalization of all experimental group 3T3 cell lines, mRNA was used. Statistical significance between different groups was determined using one-way analysis of variance (ANOVA). The list of primers for quantitative RT-PCR can be found in Table 2.

### 4.9. Western-Blot

Tissue samples (50 mg) were solubilized in Ripa Buffer (89900, Thermo Fisher, Waltham, MA, USA) with addition of EASYpack protease inhibitors (5892970001, Roche). Protein concentration in supernatants after 14,000 rpm centrifugation was determined using the BCA method. 25 mkg of protein were separated on 8% SDS-PAGE with subsequent blotting on nitrocellulose membrane (BIO-RAD) using Trans-Blot^®^ Turbo™ Transfer System (Bio-Rad) with subsequent immersion in 5% skimmed milk in TBST buffer. 

Primary ab: 1:1000-FSTL1, (BAF1738, R&D System); GAPDH (32233, Santa-Cruz). Secondary ab: 1:10000-anti-Mouse IgG (H+L) Secondary Antibody (115-005-003, Jackson ImmunoResearch), anti-Rabbit IgG (H+L) Secondary Antibody (111-035-144, Jackson ImmunoResearch). Signal was revealed by the ECL method following the HRP reaction.

### 4.10. Statistical Analysis

Data are presented as mean value ± SD. Statistical analyses were performed using a one-way ANOVA a Kolmogorov-Smirnov test for rank as appropriate using Graph Pad Prism 8 software. Differences were considered significant at values of *p* < 0.05.

## 5. Conclusions

In conclusion, we confirm the anti-fibrotic action of 4MU in mouse CCl_4_-induced liver fibrosis. We demonstrate that this compound affects basic profibrotic processes. Mainly, it inhibits ECM deposition by directly affecting the production of its main components Col1a and HA. 4MU prevents the activation of HSCs and their migration towards the sites of fibrogenesis. It also alters the distribution of macrophages and changes their expression pattern towards the regenerative phenotype. 4MU downregulates the expression and protein level of the potent activator of the TGF-beta pathway FSTL1, which was shown to have strong profibrotic action. 

Our data warrants investigation of 4MU for its anti-fibrotic action in clinical trials. Availability of the approved drug, hymecromone, containing 4MU as an active compound, will facilitate clinical studies for the repurposing of hymecromone for the treatment of liver fibrosis.

## Figures and Tables

**Table 1:** Thresholded Manders’ coefficient for paired markers.

**Macrophages (Man HAS2; F4/80)**		
	**M (HAS2)**	**M (F4/80)**
Control	0.39 ± 0.17	0.2 ± 0.12
4MU	0.53 ± 0.2	0.68 ± 0.16 ***
CCL_4_	0.7 ± 0.13 *	0.38 ± 0.12
CCL_4_/4MU	0.32 ± 0.08 ^##^	0.36 ± 0.09
**HSCs (Man HAS2; GFAP)**		
	**M(HAS2)**	**M(GFAP)**
Control	0.55 ± 0.07	0.10 ± 0.02
4MU	0.27 ± 0.07 ***	0.10 ± 0.02
CCL_4_	0.37 ± 0.07 **	0.11 ± 0.02
CCL_4_/4MU	0.36 ± 0.05	0.15 ± 0.02 ^#^

*** *p* < 0.001 (compared to control), ** *p* < 0.01 (compared to control), * *p* < 0.05 (compared to control). ^##^ *p* < 0.01 (compared to CCL4), ^#^ *p* < 0.05 (compared to CCL4).

**Table 2:** List of primers for Quantitative RT-PCR.

Gene	Direction	Sequence
*GAPDH*	Foward	5′-AGGTCGGTGTGAACGGATTTG-3′
	Reverse	5′-TGTAGACCATGTAGTTGAGGTCA-3′
*Acta2*	Forward	5′-CTGAAGAGCATCCGACACT-3′
	Reverse	5′-AGCCTGAATAGCCACATACAT-3′
*Col1a*	Forward	5′-CCGCAAAGAGAGTCTACATGTC-3′
	Reverse	5′-CTGACTTCAGGGATGTCTTC-3′
*Hyal1*	Forward	5′-AACAAGTACCAAGGAATCAT-3′
	Reverse	5′-GAGAGCCTCAGGATAACT-3′
*Fstl1*	Forward	5′-GTGAGATCCTAGACAAGTA-3′
	Reverse	5′-TCTCCTGATCTGCATAAG-3′

## Supplementary Materials

Supplementary materials can be found at https://www.mdpi.com/1422-0067/20/24/6301/s1.
