# Supplementary material for: 4-methylumbelliferone Prevents Liver Fibrosis by Affecting Hyaluronan Deposition, FSTL1 Expression and Cell Localization
Source: Int J Mol Sci. 2019 Dec 13;20(24):6301. doi: 10.3390/ijms20246301 (PMC6941058; doi:10.3390/ijms20246301)
Supplement: Supplementary file 1 [file ijms-20-06301-s001.zip › ijms-666808-supplementary/ijms-666808-supplementary.docx]

**Supplementary:**

**
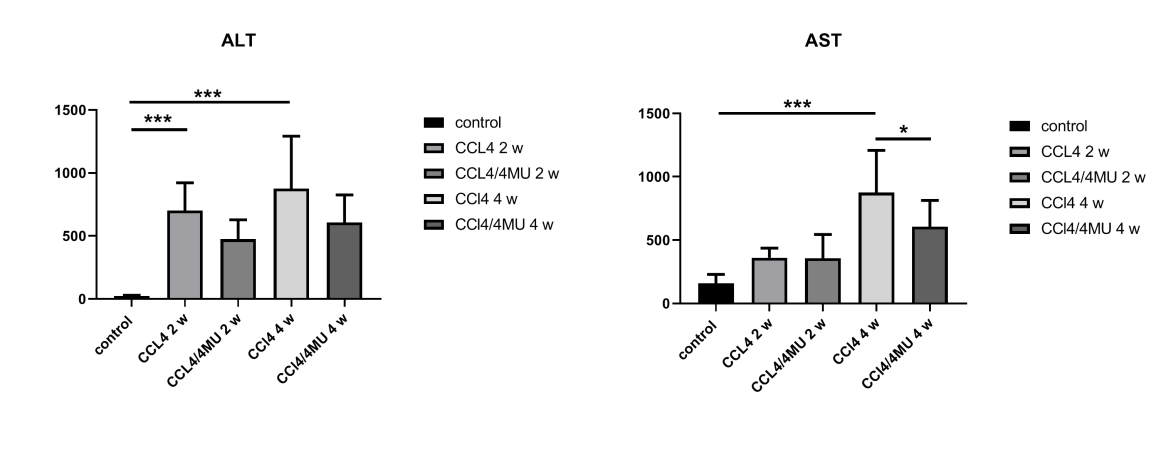
**

**Figure S1.** ALT/AST level changes during CCl_4_ or CCl_4_/4MU exposure. Data represented in mean ± SD, n=8 for CCL_4_ and CCL_4_/4MU treated groups, n=5 for control group.


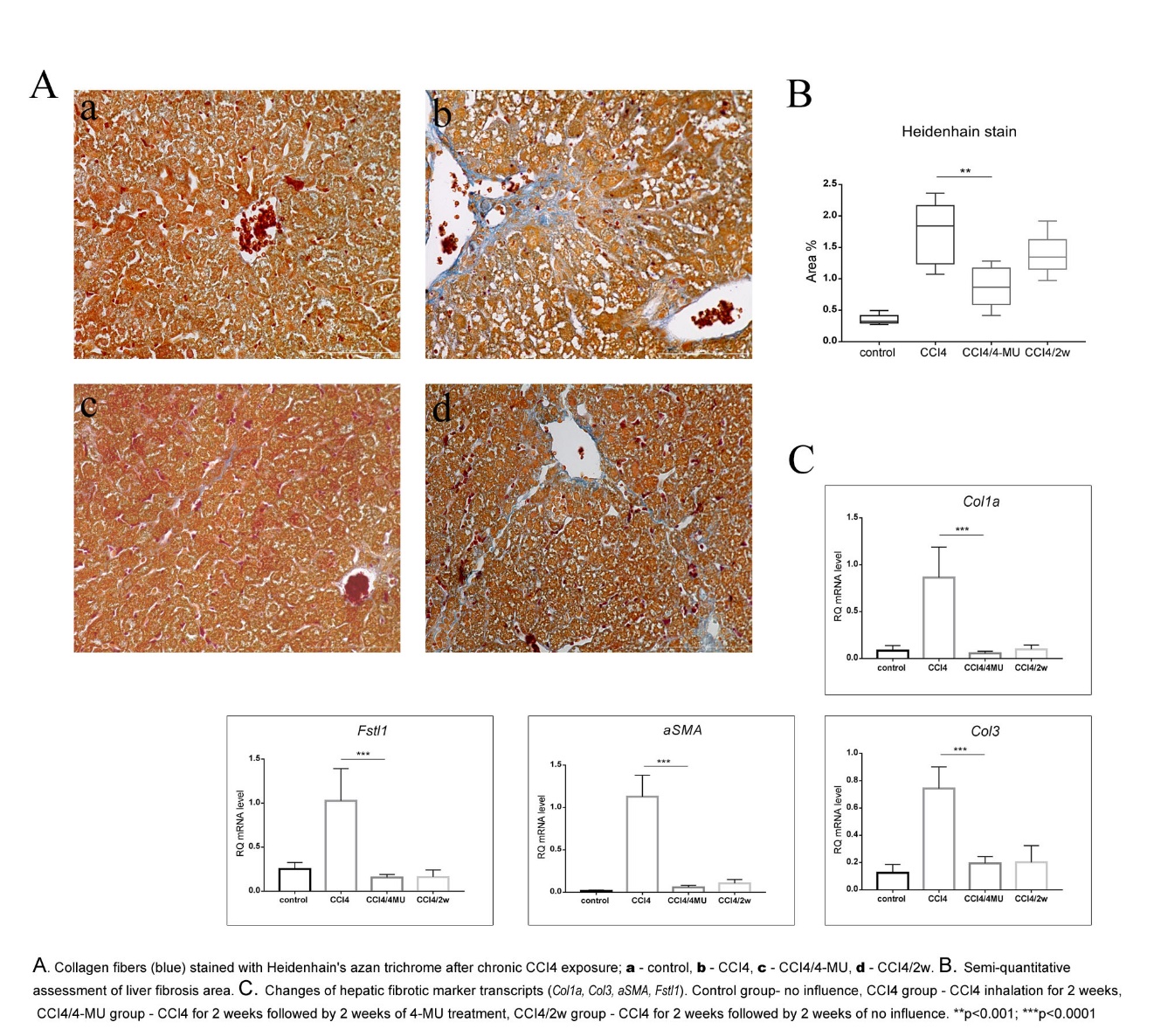


**Figure S2.** 4MU treatment intensifies fibrosis resolution. 4MU treatment was started after 2 weeks of CCl_4_ exposure and continued for 2 weeks. A. Collagen fibers (blue) stained with Heidenhain’s azan trichrome; **a**- control, b – CCl_4_ 2 week, c – CCL_4_/4MU : CCl_4_ 2 week followed by 2 week of 4MU only treatment, d – CCl_4_/2w : CCl_4_ for 2 week followed by no treatment for 2 additional weeks. B – Quantitative assessment of liver fibrosis. C – Q-PCR analysis of hepatic fibrosis marker transcripts. Data presented as mean ± SD **p =0.001, ***p=0.0001

**
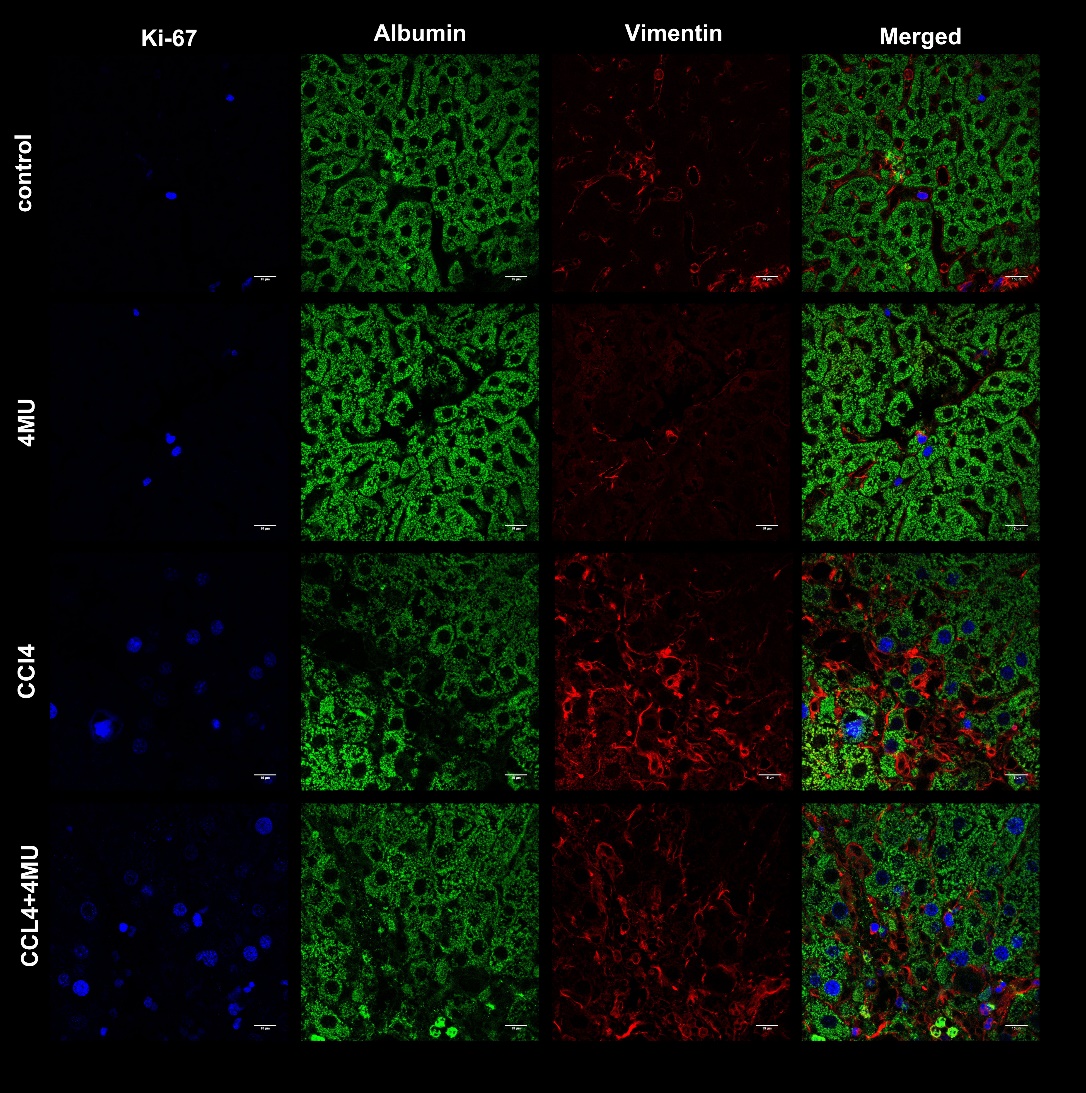
**

**Figure S3.** Triple staining for Ki-67 (proliferating cells), Albumin (hepatocytes) and Vimentin (HCSs).

Evaluation of Vim+/Ki-67 positive cells was done manually in ImageJ. For statistical analysis, each experimental groups include 3 samples (for control and 4MU treatment) and 5 samples (for CCL4 and CCl4/4MU treated samples), 9 z-stacks 50 um in dept with 2 um z-step were taken from each sample. Each z-stack was analyzed manually, by calculating Vim+/Ki-67+ cells, located in liver parenchyma. One-way ANOVA was performed in GraphPad Prism 8.0 software.
